# Interfacial Effects of Nonionic and Anionic Surfactants on the Colloidal Stability of Graphene and Al_2_O_3_ Nanoparticles in Jet-A/Sustainable Aviation Fuel Blends

**DOI:** 10.1021/acsomega.6c01909

**Published:** 2026-06-12

**Authors:** Yasmin Wadzer, Hussin Mamat, Syed Afdhal Sayed Ghazali, Nurul Musfirah Mazlan

**Affiliations:** a School of Aerospace Engineering, Tuanku Syed Sirajuddin Engineering Campus, 65270 Universiti Sains Malaysia, Kampus Kejuruteraan, Nibong Tebal, Pulau Pinang 14300, Malaysia; b Petronas, Level 65, Tower 2, Kuala Lumpur 50088, Malaysia

## Abstract

Sustainable aviation fuel (SAF), such as hydrotreated vegetable oil (HVO) mixed with Jet-A, has attracted increasing interest as an alternative aviation fuel to reduce aircraft-related emissions and support long-term decarbonization targets. The incorporation of nanoparticles has been reported to enhance the physicochemical and thermophysical performance of the blends. However, the practical implementation of nanoparticle-based SAF blends is constrained by challenges associated with the long-term colloidal stability in nonpolar fuel systems. In this study, the interfacial effects of nonionic and anionic surfactants on the colloidal stability of graphene (Gr) and aluminum oxide (Al_2_O_3_) nanoparticles dispersed in Jet-A/HVO blends were systematically investigated. Cetyltrimethylammonium bromide (CTAB), sorbitan monooleate (SPAN 80), and sodium dodecylbenzenesulfonate (SDBS) were evaluated at surfactant-to-nanoparticle ratios of 1:0.5, 1:1, and 1:2 to identify optimal stabilization conditions. Nanofuels were prepared using a two-step dispersion approach comprising magnetic stirring followed by ultrasonic homogenization to minimize nanoparticle agglomeration. The morphologies of Gr and Al_2_O_3_ were characterized using scanning electron microscopy. Colloidal stability was assessed through visual sedimentation observations, thermal conductivity measurements, and ζ-potential analysis. The results indicate that SPAN 80 provides superior interfacial compatibility with both nanoparticles in the fuel blends compared to CTAB and SDBS, resulting in enhanced dispersion stability. Optimal stabilization was achieved at a surfactant-to-nanoparticle ratio of 1:1 for Gr and 1:2 for Al_2_O_3_, as achieved by higher absolute ζ-potential values and improved thermophysical behavior. These findings demonstrate the critical role of surfactant chemistry and interfacial interactions in governing the colloidal stability of nanoparticle-containing sustainable aviation fuel blends.

## Graphical abstract

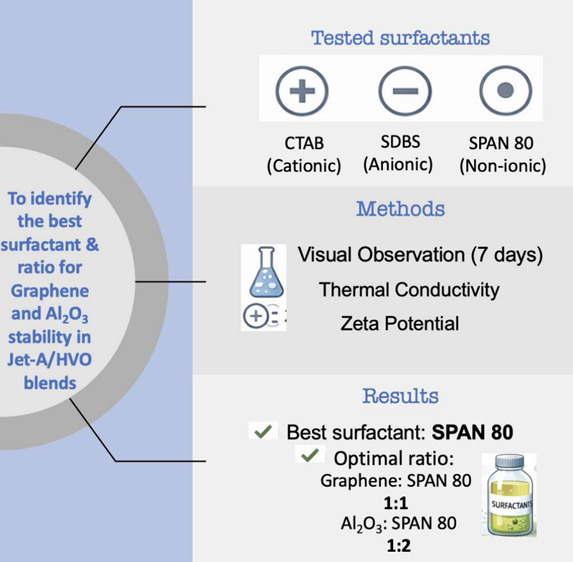

## 1 Introduction

Aviation plays a critical role in the global response to climate change. In 2018, the aviation sector was responsible for about 2.5% of global carbon dioxide emissions and 3.5% of total global warming when non-CO_2_ effects are included. By 2019, emissions from Jet-A had reached 1027 Mt CO_2_. Although aviation contributes only 12% of the combined transport sector’s CO_2_ emissions, it remains the most technologically and economically challenging sector to decarbonize.^1^ Implementation of sustainable aviation fuels (SAFs) derived from non-fossil sources is considered by numerous stakeholders as an effective approach to decarbonising aviation in the short to medium term. Furthermore, SAF has the capacity to mitigate the non-CO_2_ impacts of aviation, which have recently garnered significant attention, particularly due to their incorporation in the European Union Emissions Trading System (EU ETS) monitoring, reporting, and verification commencing in 2025.^2^

Hydrotreated Vegetable Oil (HVO) is a type of sustainable aviation fuel (SAF) that plays an important role in lowering aircraft emissions, improving fuel economy, and increasing energy security. As the aviation industry investigates alternatives to fossil-based Jet-A fuel, HVO has received interest due to its renewable nature, smaller carbon footprint, and compatibility with existing aircraft engines.^3^ HVO is produced through the hydrocracking or hydrogenation of used cooking oil. Hydrocracking uses hydrogen to break down larger molecules, whereas hydrogenation adds hydrogen to existing molecules, producing the fuel, which has a similar molecular composition to Jet-A.^4^ However, ASTM D7566 mostly regulates SAF, whereas ASTM D1655 mostly regulates conventional Jet-A/A-1. The main restriction of SAF is that it does not always meet the physical and chemical standards of Jet-A in its purest form, which is 100% unblended.^5^ The development of new fuels, such as hydrotreated vegetable oil (HVO), requires several key properties to meet ASTM D1655 specifications, including energy density and storage density for range determination, as well as freezing point and flash point as critical measures for aircraft safety. Among these properties, energy and storage densities have a significant influence on aircraft design and the flight envelope.^6^ In comparison to Jet-A, HVO has low aromatic content, whereas Jet-A contains 8% to 25% aromatics, and most SAF pathways (HEFA, FT-SPK) contain less than 0.5% aromatics. Aromatics are essential for swelling elastomer seals in older engine fuel systems to prevent leaks.^7^ Not only that, but HVO also has a lower density, where Jet-A has a minimum density of 775 kg/m^3^, while HVO usually has a density of around 750 kg/m^3^ at 15 °C. Using low-density fuel can shorten the range of an aircraft since less mass and energy can fit in the same tank volume.^8^ Furthermore, the boiling profiles of the two fuels are different. SAF is a kerosene-type fuel, and its distillation curve is frequently narrower than Jet-A’s. These variations will also affect how well some engines start up cold and how well they relight at high altitudes.^9^ Biodiesel, such as HVO, is made by transesterifying triglycerides, which are a type of fat found in food and nonedible oils. Methanol interacts with triglycerides in the presence of caustic soda as a catalyst, resulting in a mixture of fatty esters. The composition of biodiesel differs from that of hydrocarbon-based jet fuel. As a result, its blend with jet fuel must be verified against the ASTM D1655 specifications for Jet A-1 and Jet-A, respectively.^10^ Hence, additives are needed to improve the limiting properties of HVO in order to enhance its characteristics so that they closely resemble those of Jet A-1.

Recently, nanoadditives have become an option to alleviate the property limitations possessed in alternative fuels. Previous studies have demonstrated the compatibility of incorporating nanoparticles into biofuels, particularly biodiesel, because of their superior physicochemical properties. Nanoparticles possess dimensions between 1 and 100 nm and are synthesized from metals, metal oxides, or other inorganic materials. Nanoparticles are produced from pure metals (or base metals) or their compounds (often oxides) through the alteration of the larger macroscopic structure of the base metal or its compound into a configuration that includes nanoscale particles or nanoparticles. Nanoparticles facilitate atomization, evaporation, and combustion in various approaches.

The addition of nanoparticles enhances combustion efficiency by increasing the surface area-to-volume ratio, thereby improving air–fuel mixing and promoting more complete combustion. Moreover, the incorporation of nanoparticles into biofuel blends has been shown to significantly reduce harmful emissions, including carbon monoxide (CO), unburned hydrocarbons (UHC), nitrogen oxides (NO_*x*_), and particulate matter (PM). For instance, the addition of ZnO nanoparticles to waste frying oil biodiesel can decrease CO and NO_*x*_ emissions by up to 8% and 11%, respectively. This reduction is attributed to the nanoparticles’ catalytic properties, which enhance oxidation reactions and stabilize flame temperature variations.^11^ Adding a nanoparticle lowers the blend’s viscosity and raises the cetane number. This makes the ignition delay shorter and the heat transfer to fuel droplets better. Consequently, the nanoparticles enhanced fuel atomization, penetration, and thermal energy transfer. These factors contribute to a reduction in the ignition delay period, resulting in improved combustion properties when nanoparticles are present in the fuel.^12^ There are different types of nanoparticles, including metal-based, nonmetal-based, carbon-based, and ceramic nanoparticles. Metal-based nanoparticles, including titanium dioxide (TiO_2_), aluminum oxide (Al_2_O_3_), zinc oxide (ZnO), and cerium oxide (CeO_2_), have emerged as key additives in biodiesel, enhancing its combustion efficiency, fuel stability, and emission characteristics. Their catalytic properties and high surface-area-to-volume ratios significantly enhance the thermophysical behavior of fuels, making them effective in addressing the inherent limitations of biodiesel.^11^ Furthermore, owing to their high reactivity and low production costs, aluminum particles have been combined with various liquid fuels and tested across a wide range of operating conditions.

Not only metal-based nanoparticles but also significant focus has been placed on carbon-based nanomaterials, such as graphene/graphite, carbon nanotubes, activated carbon nanoparticles, and acetylene black, owing to their exceptional thermal properties. These characteristics make them outstanding candidates for the development of nanofluids and nanolubricants.^13^ Carbon-based nanomaterials such as graphene exhibit excellent thermal conductivity and a large specific surface area. These properties boost the combustion efficiency and help lower emissions in biodiesel use. Additionally, these nanoparticles enhance fuel atomization and accelerate combustion cycles, resulting in improved engine performance and reduced emissions.^11^ Graphene (Gr) is a popular carbon-based material due to its high thermal conductivity (up to 5 000 W/mK) and unique shape-driven properties. As a single layer of carbon atoms, it can have varying surface functionalities and impact performance, which may enhance the efficiency of fuel combustion and contribute to lower emissions when used in blended jet fuels. Gr has a larger specific heat capacity and lower density than other materials, making it ideal for nanofluids or nanofuels.^14^ However, there are limited studies that have investigated the addition of Gr to the blended jet fuels. Despite the growing interest in nanofuels, studies of the dispersion stability of Gr in aviation fuel blends remain scarce. In particular, limited attention has been given to Gr nanoparticles in Jet-A and HVO fuel mixtures. This lack of systematic investigation presents a significant research opportunity to elucidate stability mechanisms, surfactant interactions, and their implications for thermal and combustion performance, thereby advancing the practical application of graphene-based nanofuels in aviation.

Inclusion of nanoparticles in the base fluid tends to agglomerate because of their high surface energy, thus reducing the stability of the liquid. Surfactants have been used in nanofluids not only to prevent agglomeration and improve nanofluid stability but also to enhance heat transfer. Several studies have examined the impact of surfactants on the nanofuels’ characteristics, including pH, thermal conductivity, specific heat, electrical conductivity, viscosity, and the mechanisms of nanofuel stability. Dispersion stability refers to the uniform suspension of nanoparticles in the base oil without sedimentation or settling owing to intermolecular interactions. Brownian motion induces continuous random movement of nanoparticles within the liquid medium, while van der Waals attractions promote particle adhesion, leading to agglomeration in the oil phase. Consequently, an increase in the cluster size of nanoparticles enhances their sedimentation rate. Hence, surfactants form charged layers surrounding the nanoparticles, preventing aggregation.^15^ However, the composition of surfactants in the solution must be carefully optimized due to complex chemical interactions, interfacial bonding behavior, and concentration-dependent effectiveness.

An insufficient ratio between the surfactant and nanoparticles results in incomplete surfactant coating and inadequate particle repulsion and agglomeration. On the other hand, a high surfactant ratio can lead to flocculation, which reduces heat transfer and thermal conductivity by increasing surfactant adsorption on the particle surfaces, thereby reducing the effective heat transfer area.^16^ Surfactants are amphipathic molecules with a nonpolar portion and a hydrophilic, polar, or ionic portion. The hydrophilic part may be anionic, cationic, nonionic, or zwitterionic. The amphiphilic nature of surfactants allows them to adsorb onto surfaces, thereby altering the surface tension and wettability.^17^

Table 1 shows the use of surfactants specifically, SPAN 80 and SDBS, for various types of nanoparticles in base fluid without comparing their agglomeration effects to those of other surfactants beforehand. As far as this paper is concerned, limited studies have investigated the effects of different types of nanoparticles on blended Jet-A fuel with sustainable aviation fuel, specifically HVO, using various types of surfactants, such as SPAN 80, CTAB, and SDBS. CTAB is a cationic surfactant with positively charged heads, while SDBS is an anionic surfactant with negatively charged heads that can be classed based on the charge on its head.^18^ Meanwhile, SPAN 80 is a nonionic surfactant; thus, it does not carry a charge on its headgroup,^19^ making it a versatile solution for oil-based systems containing both normally nonpolar and potentially polar/charged components.^20^ Considering the unique characteristics of these surfactants, it is crucial to evaluate the compatibility of CTAB, SPAN 80, and SDBS surfactants on Al_2_O_3_ and graphene in a mixture of HVO and Jet-A fuel based on visual observation, thermal conductivity, and ζ-potential. Furthermore, the dispersion stability relies on repulsive forces between scattered nanoparticles, making the dispersion method crucial. Adding an optimum surfactant avoids electrostatic repulsive forces and compensates for van der Waals attraction forces, ensuring appropriate coating; hence, the ratio of the nanoparticles to the surfactant has a strong influence on the dispersion quality and stability.^21^

**Table 1:** List of Studies on the Effect of Surfactant on Nanofuels

concentration of nanoparticles used	base fluid	surfactant	key findings	study limitations	ref.
0.25 vol % up to 3 wt % for Carbon-rich fly ash microparticles (CFA) and Single-walled carbon nanotubes (SWCNT)	Jet-A fuel	SPAN 80	SWCNTs showed superior performance, with a 13% increase at 1 vol %; CFA achieved an 8% increase at 3 vol %	while surfactants improved stability, the suspensions are still prone to sedimentation over longer periods (often a few days), limiting their use in static storage tanks without agitation	^22^
			SWCNTs caused a significant, nonlinear increase in viscosity compared to the more moderate impact of CFA		^22^
0.1 vol % of Al_2_O_3_	50% of Jet-A-1 and 50% of SPK GTL	SPAN 80	the high surface-area-to-volume ratio of Al_2_O_3_ accelerated the evaporation rate of fuel droplets, particularly for alternative fuels (SPK/GTL), which typically have lower aromatics	the study only used 0.1 wt %; it did not explore higher loadings, which might lead to nozzle clogging or significantly different atomization physics	^23^
			the addition of 0.1 vol % Al_2_O_3_ did not significantly alter the spray cone angle or the global spray structure, meaning it is compatible with existing engine hardware		^23^
0.1 vol % - 0.5 vol % of Gr	Vegetable Oil (canola, sunflower, and olive oil)	sodium lauryl sulfate	0.3 vol % was an effective threshold; exceeding this concentration led to agglomeration, which reduced the lubrication efficiency	while vegetable oil is eco-friendly, its high viscosity makes it difficult to maintain a perfectly stable graphene suspension without high-power sonication	^24^
25 mg/L, 50 mg/L, and 100 mg/L of Al_2_O_3_	Pure GTL fuel	SPAN 80	the 50 mg/L concentration was found to be the optimum concentration for improving spray characteristics without causing excessive viscosity issues	SPAN 80 provided initial stability but the Al_2_O_3_ particles began to settle after 48 h	^25^
0.1, 0.5, and 1.0 wt %. of Carbon Nanoparticle (CNP), Multiwalled carbon nanotubes (MWNCT),Gr	Jet-A	SPAN 80	small amount of nanoparticles improved thermal conductivity, with the maximum increase of 29% observed at 3% loading of multiwalled nanotubes	although higher concentrations are likely to result in higher thermal conductivities, this study did not take this into account due to rapid agglomeration and potential phase separation during measurements	^13^
25, 50, and 75 ppm of Gr	SFOME20 blend (20% of Sterculia fetida methyl ester and 80% of diesel)	QPAN 80	the optimal ratio of GNPs to SPAN 80 (1:4) is recommended for effective dispersion in biodiesel/diesel mixtures	the paper did not mention the stability period	^26^
2 and 4 wt % of Al_2_O_3_	GTL fuel	SPAN 80	the study found that the dispersion of nanoparticles in fuel resulted in consistent changes in physical properties, as stated in the literature	the inclusion of nanoparticles causes the fuel sheet to become unstable and break up into droplets fast and this leads to increased initial spray velocities and shorter breakup distances, which must be properly accounted for when evaluating the evaporation and combustion efficiency of Graphene or Alumina-based fuels	^27^
50 and 100 ppm of Al_2_O_3_	Diesel fuel	SPAN 80	while smaller nanoparticles generally provide a higher surface-area-to-volume ratio (improving combustion), they are also significantly more prone to agglomeration.	further research is needed to determine the best additive size range and concentration	^28^
0.1 and 0.2 wt % Al_2_O_3_	Bio-oil	SDBS	lowering the SDBS concentration resulted in poor nanofluid stability due to increased aggregation.	while the study demonstrates increased thermal conductivity at ambient temperature, more research is required to evaluate the thermal stability of the nanofluid under continuous heat-exchange cycles	^29^
			at an ideal SDBS concentration (0.6 wt %), better stability, improved thermal conductivity enhancement, and lower viscosity were found for both 0.1 and 0.2 wt % Al_2_O_3_		^29^
0.025–0.15 wt % of Gr	epoxidized soybean oil and polyalphaolefin 8 oil	SPAN 80	an improvement was observed on the stability upon adding 0.05 wt % of SPAN 80 to the base fuel	the optimal concentration of graphene in PAO 8 oil that minimized friction was 0.08 wt %; it was assumed that when the concentration of graphene went increased, the friction coefficient will increase; Aggregation of graphene nanoparticles could be one of the possible causes of this phenomena; furthermore, more research work might be done to uncover the cause of this	^30^
Gr	Simarouba biodiesel	SDS	adding graphene nanoparticles and adjusting injection timing reduced combustion duration while slightly increasing peak cylinder pressure at all operational loads.	future research is needed to investigate after-treatment devices (such as SCR or EGR) or secondary fuel additives that can decrease this NO_*x*_ rise without sacrificing the thermal benefits of graphene	^31^
0.5 wt % - 4 wt % of Boron	aviation turbine kerosene	SPAN 80	sorbitan oleate proved to be the most effective surfactant, with a weight ratio of boron particles to sorbitan oleate of approximately 2	the high concentration of surfactants required to stabilize boron has the potential to alter kerosene’s surface tension and flash point; According to the study, more research is needed to develop low-concentration stabilizers that provide maximum colloidal dispersion without jeopardizing the fuel’s chemical certification norms (ASTM D1655)	^32^

### 1.1 Outline of the Study: Novelty

While previous studies have reported the use of individual surfactants such as SPAN 80 and SDBS in hydrocarbon base fluids, comprehensive comparative investigations of different surfactant chemistries in blended aviation fuel systems remain limited. In particular, the interfacial interactions of cationic (CTAB), anionic (SDBS), and nonionic (SPAN 80) surfactants with graphene and aluminum oxide (Al_2_O_3_) nanoparticles in Jet-A/hydrotreated vegetable oil (HVO) blends have not been systematically evaluated. Therefore, this study aims to investigate the colloidal stability of graphene and Al_2_O_3_ nanoparticles dispersed in Jet-A/HVO blends by using varying surfactant types and surfactant-to-nanoparticle ratios. By correlating surfactant chemistry and composition with visual sedimentation behavior, thermal conductivity, and zeta potential, this work seeks to identify the most suitable surfactant and optimal ratio for each nanoparticle system, thereby providing mechanistic insight into surfactant-governed stabilization strategies for sustainable aviation fuel blends.

The outline of the present work is as follows:

Section 2 outlines the materials and methods employed in this study. The materials include the base fluids used, selected nanoparticles, and selected surfactants.This section also explains the preparation of nanofuels, the composition of nanoparticles and surfactants based on the ratio, and the methods for characterizing the stability of nanofuels.
Section 3 presents results and discussion covering the nanoparticle characterization and nanofuel stability with various surfactants and ratios. It provides a comprehensive analysis of the influence of various types and ratios of surfactants on the stability of nanofuels.

## 2 Materials and Methodology

### 2.1 Base Fluids

The SAF used in this study was HVO derived from used cooking oil obtained from SkyNRG, The Netherlands. It is produced through a hydrogenation conversion process in which oxygen molecules in the oil are removed via hydrodeoxygenation, resulting in straight paraffinic molecules. These molecules are cracked and isomerized to match the chain length of the jet fuel. HVO features a high cetane number and contains no sulfur, aromatics, or oxygen. Meanwhile, commercial aviation fuel (Jet A-1) is a fractional distillation of crude oil. This fuel consists of various hydrocarbons, including *n*-paraffins, isoparaffins, naphthenes, and aromatics. According to the ASTM D7566 specification, HVO should be combined with Jet A-1 fuel at a blend ratio of up to 50%.^33^ These combinations are safe to use in current in-flight aircraft engines without requiring further engine modifications and would not cause any fuel quality concerns. The characteristics of HVO and Jet-A are given in Table 2.

**Table 2:** Properties of HVO and Jet-A

Properties	Jet-A	HVO
Molecular formula	C_12_H_23_	C_12_H_26_
Density (kg/m^3^) @ 15 °C	800	750.3
Heat of combustion (MJ/kg)	43.031	46.025
Heating capacity (J/kgK)	2093.85	376
Enthalpy of formation (kJ/mol)	–321.70	–330.50

### 2.2 Nanoparticles

Graphene (Gr) is used in this study because of its excellent thermal conductivity properties, while Al_2_O_3_ is chosen for its low cost and high conductivity. Considering that Gr and Al_2_O_3_ are two different types of nanoparticles, it is crucial for this study to examine the particles separately and account for their distinct properties. Table 3 shows the properties of Gr and Al_2_O_3_.

**Table 3:** Properties of Used Nanoparticles

Properties	Al_2_O_3_	Gr
Molecular weight (g/mol)	101.96	12.01
Size (nm)	50	11–15
Density (g/m^3^)	4	2.27
Color	white	black
Concentration (vol %)	0.1	0.1

### 2.3 Surfactants

In this study, CTAB and SDBS were used in powder form. CTAB is a cationic surfactant with positively charged heads, whereas SDBS is an anionic surfactant with negatively charged heads. SPAN 80 is a liquid, nonionic surfactant with uncharged headgroups. SPAN 80s nonionic nature and lipophilicity (oil-loving property) also make it a versatile option for oil-based systems, including both typically nonpolar and possibly polar or charged environment components. Three different types of surfactant charges were used in this study to determine which is the most suitable for the base fluid. Three different ratios of each surfactant, which are 1:0.5, 1:1, and 1:2, were tested to identify the optimal ratio for each Gr and Al_2_O_3_ nanofuel. The properties of surfactants for each ratio are shown in Table 4. The surfactant-to-nanoparticle ratios of 1:0.5, 1:1, and 1:2 were chosen to test the transition from insufficient surface coverage to better stability. A 1:0.5 ratio was used to show how an inadequate coating affects rapid sedimentation. In contrast, a maximum ratio of 1:2 was determined since high surfactant concentrations in nonpolar systems might produce flocculation and the formation of a thermal resistance layer, reducing the heat transfer capabilities of the nanofuel. Higher ratios were avoided to avoid degrading effects since 1:1 for Gr, and 1:2 for Al_2_O_3_, provided maximum stability and thermophysical performance.

**Table 4:** Properties of Surfactants Used in This Study

Properties	CTAB	SDBS	SPAN 80
Molecular weight (g/mol)	364.45	348.5	428.6
Density (g/cm^3^) at 20 °C	2.3	0.18	1.0
Color	white	white	orange
Form	powder	powder	liquid

### 2.4 Nanofuel Preparation

The nanofuels were prepared using a two-step method. A schematic of the two-step preparation method is shown in Figure 1. Initially, an IKA C-MAG HS 7 magnetic stirrer was used to agitate the base oil, nanoparticles, and surfactant for 20 min at a speed of 1000 rpm. Furthermore, a Hielscher UP400 St ultrasonic homogenizer (sonicator) was used for 45 min at 70% amplitude to disperse clumped nanoparticle clusters and produce more homogeneous samples. The sample temperature began to rise as the procedure commenced and eventually reached 70 °C; however, the sample was maintained in an ice–water bath. The viscosity of the base oil decreased at this temperature. Consequently, the sonication process was improved.

**Figure 1:**
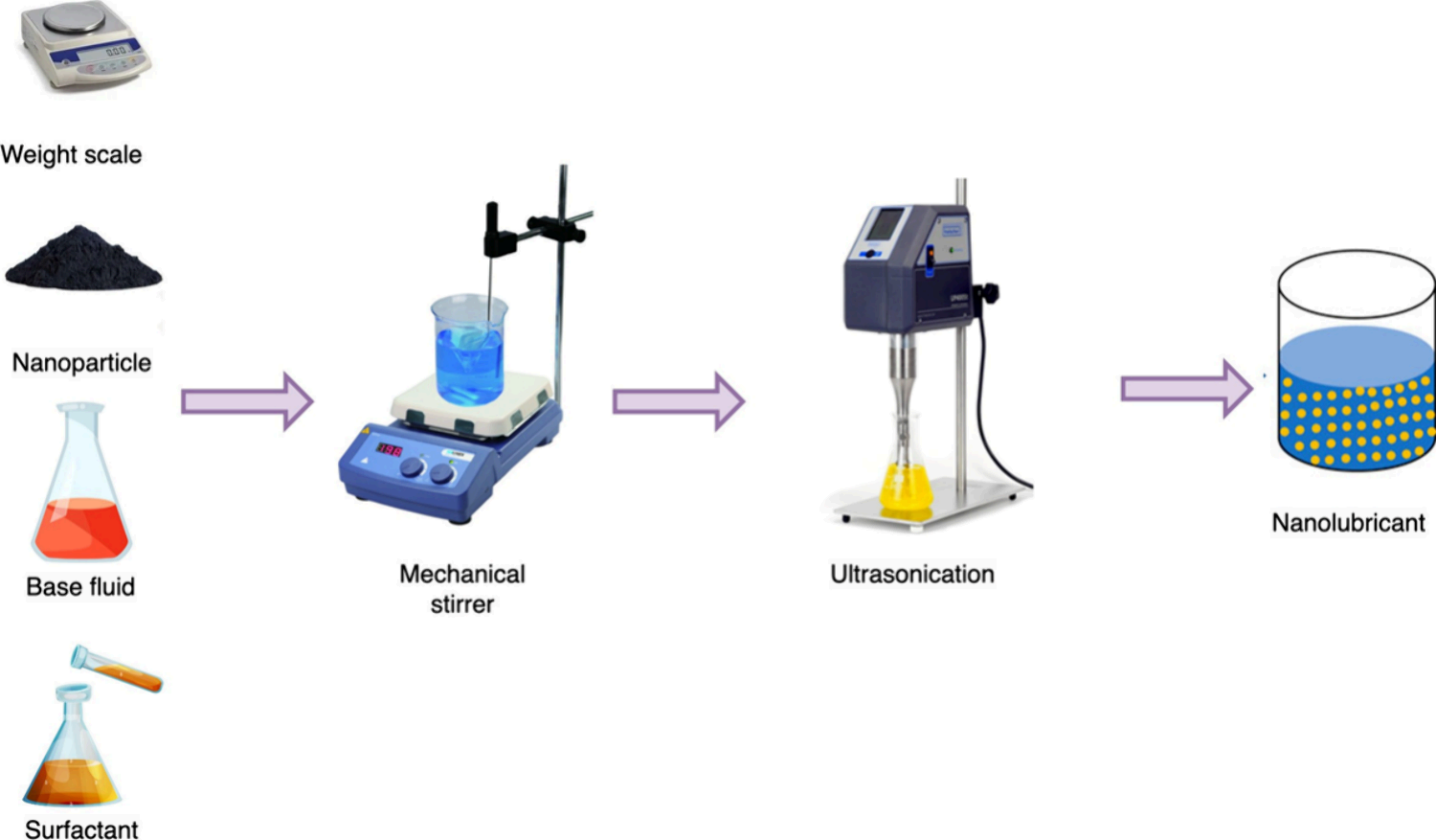
Schematic diagram of a two-step method for nanolubricant preparation.^34^ Reprinted with permission from ref 34. Copyright 2024 Elsevier. License Number: 6247060542979.

Table 5 shows 18 fuel samples considered in this study. The fuel samples contained a combination of Jet-A/HVO blend, nanoparticles, and various nanoparticle-to-surfactant ratios used in this study. A concentration of nanoparticles was taken as 0.1 vol % for all samples, while the ratio of nanoparticles to surfactants was varied at 1:0.5, 1:1, and 1:2. All fuel samples were given code names to identify the sample and its contents. This code was consistently used throughout the study.

**Table 5:** Samples Used in the Study

		Weight of nanoparticles		Weight of surfactants		
No.	Code name	Gr vol% (grams)	Al_2_O_3_ vol% (grams)	CTAB (grams)	SDBS (grams)	SPAN 80 (grams)
1	GR-JETA/HVO(0.1)CT (1:0.5)	0.0908		0.022		
2	GR-JETA/HVO(0.1)SD (1:0.5)	0.0908			0.0200	
3	GR-JETA/HVO(0.1)SP (1:0.5)	0.0908				0.0198
4	GR-JETA/HVO(0.1)CT (1:1)	0.0908		0.0445		
5	GR-JETA/HVO(0.1)SD (1:1)	0.0908			0.0400	
6	GR-JETA/HVO(0.1)SP (1:1)	0.0908				0.0396
7	GR-JETA/HVO(0.1)CT (1:2)	0.0908		0.0889		
8	GR-JETA/HVO(0.1)SD (1:2)	0.0908			0.0800	
9	GR-JETA/HVO(0.1)SP (1:2)	0.0908				0.0793
10	Al_2_O_3‑_JETA/HVO(0.1)CT (1:0.5)	0.0908		0.0220		
11	Al_2_O_3_-JETA/HVO(0.1)SD (1:0.5)		0.1598		0.0200	
12	Al_2_O_3_-JETA/HVO(0.1)SP (1:0.5)		0.1598			0.0198
13	Al_2_O_3_-JETA/HVO(0.1)CT (1:1)		0.1598	0.0445		
14	Al_2_O_3_-JETA/HVO(0.1)SD (1:1)		0.1598		0.0400	
15	Al_2_O_3_-JETA/HVO(0.1)SP (1:1)		0.1598			0.0396
16	Al_2_O_3_-JETA/HVO(0.1)CT (1:2)		0.1598	0.0889		
17	Al_2_O_3_-JETA/HVO(0.1)SD (1:2)		0.1598		0.0800	
18	Al_2_O_3_-JETA/HVO(0.1)SP (1:2)		0.1598			0.0793

### 2.5 Morphology of Nanoparticles

The morphology of Gr and Al_2_O_3_ was examined with scanning electron microscopy (SEM, FEI VERIOS 460L) using an extremely high-resolution (XHR) with field emission with an accelerating voltage of 5 kV. Imaging was conducted at different magnifications, from 3k× to 100k× for Gr and 15k× to 200k× for Al_2_O_3_, to capture both micro- and nanoscale features.

### 2.6 Characterization of Dispersion Stability

Dispersion stability was evaluated qualitatively and quantitatively through visual observation, thermal conductivity, and ζ-potential.

#### 2.6.1 Visual Observation

A visual observation method was employed to assess the sedimentation of the nanoparticles in the base fluid. The samples were observed for a 7 day period. The ratios of surfactant to nanoparticle were varied to 1:0.5, 1:1, and 1:2, with a fixed concentration of 0.1 vol% for each sample. The study employed a box with an LED-lit background to monitor the images of the aggregation and sedimentation in the sample bottles. The setup for this method is listed in Figure 2. These aggregates were visible through imaging but not with the naked eye. An external black box evenly scattered the light and prevented external light and reflections. The experiment aimed to determine whether sonication was successful; identify which sample had a reliable surfactant type and concentration, which would last longer; and detect any other phenomena, such as color change or large agglomeration, at different surfactant ratios.

**Figure 2:**
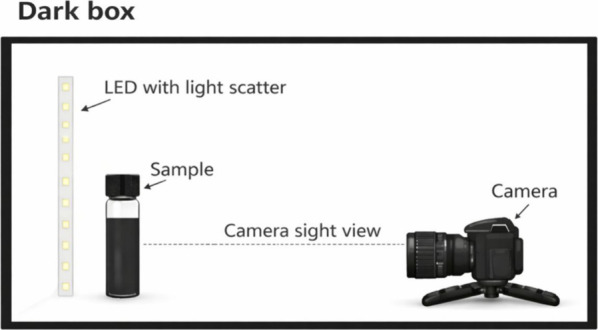
Schematic diagram of setup used for the visual observation method.

#### 2.6.2 Thermal Conductivity

The stability of the nanofuel samples was further tested by measuring their thermal conductivity using a KD2 Pro Thermal Properties Analyzer with a KS-1 sensor, as it is recommended for liquid and nanofuel samples. Only samples that remained stable for up to 3 days, as observed visually, were selected for evaluation. Thermal conductivity was used as an additional indicator of stability, as stable dispersions maintain consistent thermal properties, whereas unstable samples, due to aggregation or sedimentation, often show reduced or variable thermal conductivity. Brownian motion, the main mechanism controlling the thermal behavior of fluid-nanoparticle dispersion, is one of several factors contributing to the increase in effective thermal conductivity. According to an alternative theory, the liquid molecules surrounding the nanoparticles create layered structures, called nanolayers. By acting as a thermal bridge between the bulk liquid and the nanoparticles, these layered structures improve the thermal conductivity. Moreover, phonons that emerge at random, move in random directions, and are scattered by flaws or collisions with one another carry heat in crystalline materials. Moreover, it is shown that particle clustering changed the effective thermal conductivity.^35^ In this study, measurements were performed in triplicate (*n* = 3); the data points represent the mean values, and the error bars indicate the standard deviation of the three independent readings.

#### 2.6.3 Zeta Potential

Zeta potential is another method used to determine the stability of the nanoparticles and surfactant in the blended fuel. The zeta potential is the electrostatic repulsion between the surface of particles and the stationary layer of fluid and provides the most precise depiction of nanofluid stability. The zeta potential test was performed on all samples using a Malvern Zetasizer zeta potential analyzer. The zeta potential measurement has been repeated and recorded three times, and the average of the zeta potential value is recorded. The error bars on the graph represent the standard deviation of the three readings. Referring to Table 6, the zeta potential value of more than +40 mV and less than −40 mV is considered stable, whereas a lower value of zeta potential determines lower nanofluid stability.^36^ Zeta potential is an important indication of electrokinetic potential in colloidal systems. While a threshold of ±30 mV is widely stated as the border for stability in much colloidal research, this work employs a stricter criterion of ±40 mV to ensure high-degree long-term stability, as indicated by^36^ in the context of ceramic nanofluids. Values above ± 40 mV indicate a high potential barrier that successfully prevents nanoparticle aggregation through strong electrostatic repulsion. This is especially important in the low-dielectric environment of aviation fuel mixes, where maintaining stability is crucial to prevent issues such as sedimentation and loss of performance in fuel systems.

**Table 6:** Stability of Colloids to Zeta Potential^36^

Stability characteristics	Average zeta potential (mV)
Rapid coagulation or flocculation	from 0 to ±5
Incipient instability	from ±5 to ±30
Moderate instability	from ±30 to ±40
Good stability	from ±40 to ±60
Excellent stability	more than ±60

## 3 Results and Discussion

### 3.1 Nanoparticles SEM Images

Figure 3 compares the morphological characteristics of graphene (Gr) and alumina (Al_2_O_3_) powders as observed through SEM imaging. Gr has thin and flaky sheets with large exposed surface areas. The sheets stack on each other. The morphology indicates that Gr possesses an ultrathin layered structure with a high specific surface area. Conversely, Al_2_O_3_ powder shows irregular, nearly spheroidal particles with thicker dimensions and reduced exposed surface area compared to that of Gr. This suggests that Al_2_O_3_ exhibits a denser morphology with a lower surface reactivity.

**Figure 3:**
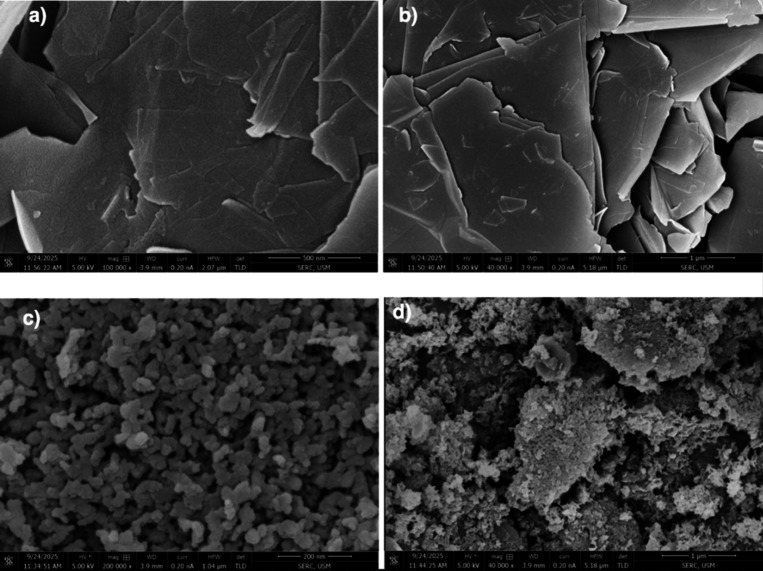
SEM images of: (a) Gr powder (scale bar 500 nm, 100k× magnification); (b) Gr powder (scale bar 1 μm, 40k× magnification); (c) Al_2_O_3_ powder (scale bar 200 nm, 200k× magnification); and (d) Al_2_O_3_ powder (scale bar 1 μm, 40k× magnification).

### 3.2 Visual Observation

Figure 4 shows the visual observation of Gr and Al_2_O_3_ when dispersed in a Jet-A/HVO fuel mixture with different surfactants (SPAN 80, SDBS, and CTAB) at a 1:0.5 nanoparticle-to-surfactant ratio observed over 7 days. Sample (a) in the figure represents pure Jet-A blended with HVO. Samples (b) to (d) in the figure represent samples comprising Gr with SPAN 80, SDBS, and CTAB, respectively. Samples (e) to (g) represent samples of Al_2_O_3_ with SPAN 80, SDBS, and CTAB, respectively. The sedimentation patterns in Figure 4 differ from those in Figure 5. Figure 4 shows that the sedimentation patterns were very different on day 1 depending on the type of surfactant. Samples (b) and (c), which included SPAN 80 and SDBS, respectively, had a lot of initial dispersion and no visible sediment at the bottom. Other samples, on the contrary, showed phase separation in the early stages. At the end of day 1, a clear layer of sediment could be seen at the bottom of the glass tube. This was because some of the nanoparticles were still suspended in the bulk liquid, which made some samples look grayish. This means that SPAN 80 and SDBS slow down agglomeration at first, but they do not totally stop larger clusters from settling in the nonpolar Jet-A/HVO medium due to gravity. The reason for the fast sedimentation is the non-optimal ratio of the surfactant to the nanoparticle. At a nanoparticle-to-surfactant ratio of 1:0.5, there are insufficient surfactant molecules to cover the total surface area of all of the nanoparticles in suspension. Consequently, a considerable proportion of the nanoparticle surfaces remained uncoated or were only partially coated. These exposed portions retain their strong attractive forces, such as Van der Waals and polar interactions. When these nanoparticles with insufficient coatings meet due to Brownian motion, their exposed surfaces come into contact. Strong attractive forces outweigh the weak or absent repulsive forces of the inadequate surfactant layer, resulting in rapid and irreversible aggregation, and the nanoparticles will clump together.^16^

**Figure 4:**
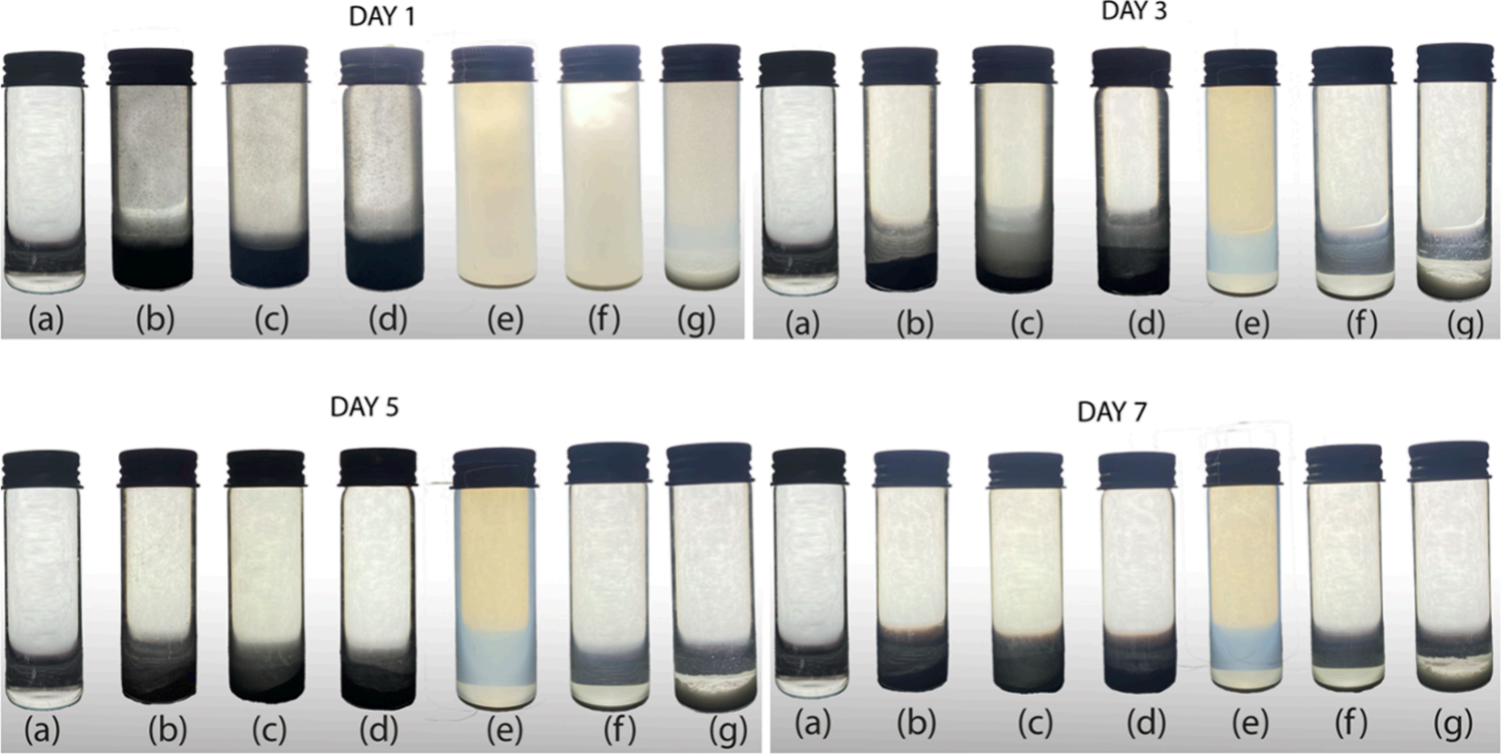
Visual observation of Gr and Al_2_O_3_ nanolubricants with surfactants with a ratio of 1:0.5: (a) pure mixture of HVO & Jet-A, (b) GR-JETA/HVO(0.1)­SP, (c) GR-JETA/HVO(0.1)­SD, (d) GR-JETA/HVO(0.1)­CT, (e) Al_2_O_3_-JETA/HVO­(0.1)­SP, (f) Al_2_O_3_-JETA/HVO­(0.1)­SD, and (g) Al_2_O_3_-JETA/HVO­(0.1)­CT from day 1 to day 7.

**Figure 5:**
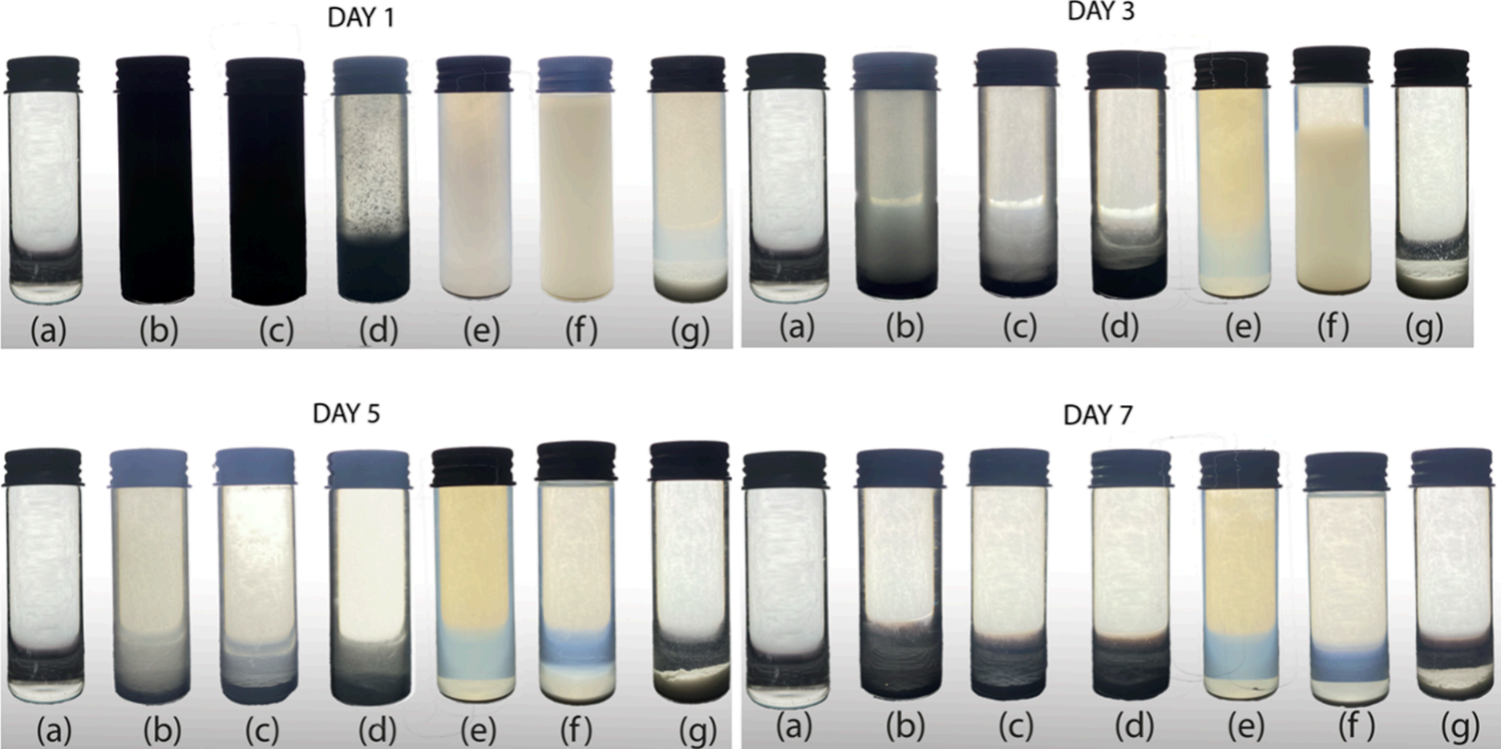
Visual observation of Gr and Al_2_O_3_ nanolubricants with surfactants with a ratio of 1:1: (a) pure mixture of HVO & Jet-A, (b) GR-JETA/HVO(0.1)­SP, (c) GR-JETA/HVO(0.1)­SD, (d) GR-JETA/HVO(0.1)­CT, (e) Al_2_O_3_-JETA/HVO­(0.1)­SP, (f) Al_2_O_3_-JETA/HVO­(0.1)­SD, and (g) Al_2_O_3_-JETA/HVO­(0.1)­CT from day 1 to day 7.

Figure 5 shows visual observation for identifying the surfactant in the Gr and Al_2_O_3_ mixtures with a 1:0.5 nanoparticle-to-surfactant ratio in a mixture of HVO and Jet-A fuel over 7 days. The sedimentation patterns in Figure 4 differ from those in Figure 5. On day 1, samples with SPAN 80 and SDBS appeared well-dispersed without any sedimentation observed, indicating effective initial dispersion of nanoparticles. On the other hand, the sample with the CTAB surfactant sedimented rapidly for both Gr and Al_2_O_3_. Differences in stability are further noticed as time emerges. On day 3, sedimentation of all Gr samples began to exhibit sedimentation except for Gr with SPAN 80, which exhibited a grayish color, indicating that the Gr remained intact with the base fluid. In contrast, Gr with SDBS and CTAB displayed a clear coloration, as all nanoparticles were sedimented, allowing more light to penetrate the glass bottle. For the Al_2_O_3_ samples (Figure 5e–g), the suspension containing SPAN 80 (Figure 5(e)) remained the most stable from day 1 to day 7. After day 7, the sedimentation SPAN 80's strength lies in its nonionic nature and lipophilicity (oil-loving tendency), making it a versatile solution for oil-based systems that comprise both typically nonpolar and potentially polar/charged components.^20^ In addition, nonionic surfactants have a hydrophilic head and hydrophobic tail, which prevents them from dissociating in water. Polyethoxylated nonionic surfactants are derived from ethylene oxide condensation. The addition of a nonionic surfactant to lubricating oil is determined by the hydrophobic–hydrophilic balance (HLB). The HLB value varies between 1 and 20. Surfactants with HLB values above 10 are hydrophilic, whereas those with HLB values below 10 are hydrophobic.^15^ Elumalai et al.^37^ used SPAN 80 as a surfactant to stabilize a mixture of biodiesel and diesel owing to its HLB value. When selecting a nonionic surfactant, it is important to consider the HLB of the emulsifier’s chemical structure and properties. Hydrophilic (high HLB value) and lipophilic (low HLB value) surfactants are commonly used to emulsify oil-in-water and water-in-oil systems. Lipophilic and hydrophilic emulsifiers have HLB values ranging from 4 to 8 and 9 to 13, respectively.^38^ In simple terms, low HLB values (e.g., 3–6) indicate that a lipophilic (oil-loving) surfactant is commonly used to stabilize water-in-oil (W/O) emulsions, in which water droplets are dispersed in a continuous oil phase. High HLB values (e.g., 8–18) indicate that a more hydrophilic (water-loving) surfactant is often employed to stabilize oil-in-water (O/W) emulsions, in which oil droplets are spread throughout a continuous water phase. SPAN 80 has a relatively low HLB value, typically around 4.3–4.7. A surfactant with a significant affinity for both oil and particle surfaces is required to disperse solid nanoparticles into the oil phase. A low HLB surfactant (oil-loving, such as SPAN 80) is crucial because it ensures that the surfactant is highly soluble in the oil mixture. If the surfactant is not soluble in oil, it is unable to properly reach the nanoparticle surface, adsorb, and stabilize. Once adsorbed, the lipophilic (oil-loving) tails of the low HLB surfactant extend into the continuous oil phase, forming a steric barrier that prevents the nanoparticles from clumping together. This confirms its strong lipophilic nature, ensuring that the surfactant was well dissolved in the mixture of HVO and Jet-A fuel.

Visual observations for the identification of the surfactant in Gr and Al_2_O_3_ mixtures with a 1:2 nanoparticle-to-surfactant ratio in a mixture of HVO and Jet-A fuel over 7 days are presented in Figure 6. On the basis of Figure 6, at the 1:2 surfactant-to-nanoparticle ratio, the Al_2_O_3_ samples (e) and (f) showed the best stability on day 1, keeping a consistent opaque look. The other samples, on the other hand, showed obvious sedimentation after 24 h. This revealed that even a larger concentration of surfactant was not enough to overcome the high surface energy of the nanoparticles in the low-viscosity fuel blend for these specific combinations. The zeta potential data that are presented in Section 3.4 show that samples (e) and (f) were more stable than the other samples, which is consistent with this visual evidence.

**Figure 6:**
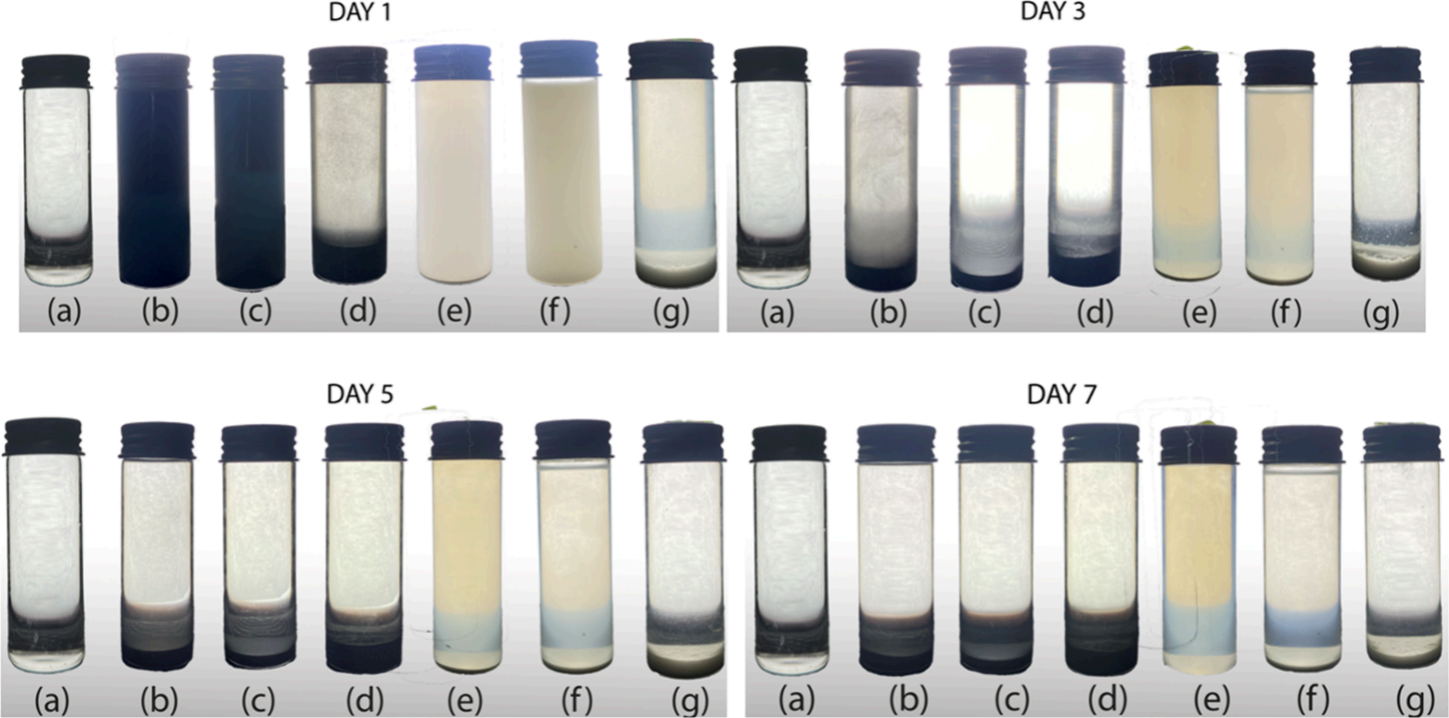
Visual observation of Gr and Al_2_O_3_ nanolubricants with surfactants with a ratio of 1:2: (a) pure mixture of HVO & Jet-A, (b) GR-JETA/HVO(0.1)­SP, (c) GR-JETA/HVO(0.1)­SD, (d) GR-JETA/HVO(0.1)­CT, (e) Al_2_O_3_-JETA/HVO­(0.1)­SP, (f) Al_2_O_3_-JETA/HVO­(0.1)­SD, and (g) Al_2_O_3_-JETA/HVO­(0.1)­CT from day 1 to day 7.

In addition, for the observations that have been examined, there are a lot of studies^13,23,25–27,39^ that have identified that SPAN 80 can stabilize the Gr and Al_2_O_3_ in nanofuels in a specific period. SPAN 80 has a long oleate (hydrophobic) tail that is highly soluble and compatible with nonpolar HEFA and the hydrocarbon components of Jet-A. The forces and charges involved in surfactant aggregation in nonaqueous solvents (such as oils and fuels) differ considerably from those in water-based systems. The hydrophilic effect of SPAN 80 lowers the unfavorable interactions between the hydrophilic part of SPAN 80 and the nonpolar solvent molecules and concomitantly increases the interactions between the hydrophilic groups, which is the primary driving force for the micelle formation in nonpolar solvents.^40^ This makes SPAN 80 compatible with a mixture of HVO and Jet-A fuel, and it remains intact for a few days.

Figures 4–6 show that CTAB with both Gr and Al_2_O_3_ was sedimented even on day 1 for all ratios. The same observation was reported in a study^41^ where CTAB has poor stability in the dispersion system consisting of calcite in white oil due to the crystal of CTAB, which leads to an increase in solid phase content in the dispersion system. Meanwhile, SPAN 80 was in the oil phase, and SDBS was in powder form, which allowed both surfactants to dissolve easily in oil and settle more slowly. Furthermore, ionic surfactants such as CTAB and SDBS consist of a highly polar/charged headgroup and a long, nonpolar hydrocarbon tail, and the headgroup carries a full or partial formal charge, creating a strongly localized electric field.^42^ Pollard et al.^43^ discovered that CTAB (a cationic surfactant) had a low solubility in the less polar solvents due to its ionic nature. Meanwhile, the anionic surfactants in the mentioned study were soluble in all solvents except toluene because of the polarity of their headgroups. Toluene is a nonpolar solvent, similar to the solvent used in the current study, which was a mixture of Jet-A and HVO fuel. This demonstrates that even for an anionic surfactant, the polarity of its headgroup is a crucial factor that limits its solubility in a nonpolar solvent. According to the cited study, anionic surfactants have unfavorable interactions between the polar head groups and the nonpolar solvent, causing the surfactant molecules to minimize contact with the solvent by aggregating rather than dissolving. Anionic and cationic surfactants only partition into more polar solvents, implying their poor solubility in nonpolar or less polar environments.

### 3.3 Thermal Conductivity

Figure 7 shows thermal conductivities of Gr and Al_2_O_3_ with SPAN 80 at nanoparticle-to-surfactant ratios of 1:1 and 1:2. Only these samples were considered due to their capability to remain intact up to 3 days using the visual observation method compared to other fuel samples, indicating the compatibility of SPAN 80 as the most suitable surfactant for both Gr and Al_2_O_3_.

**Figure 7:**
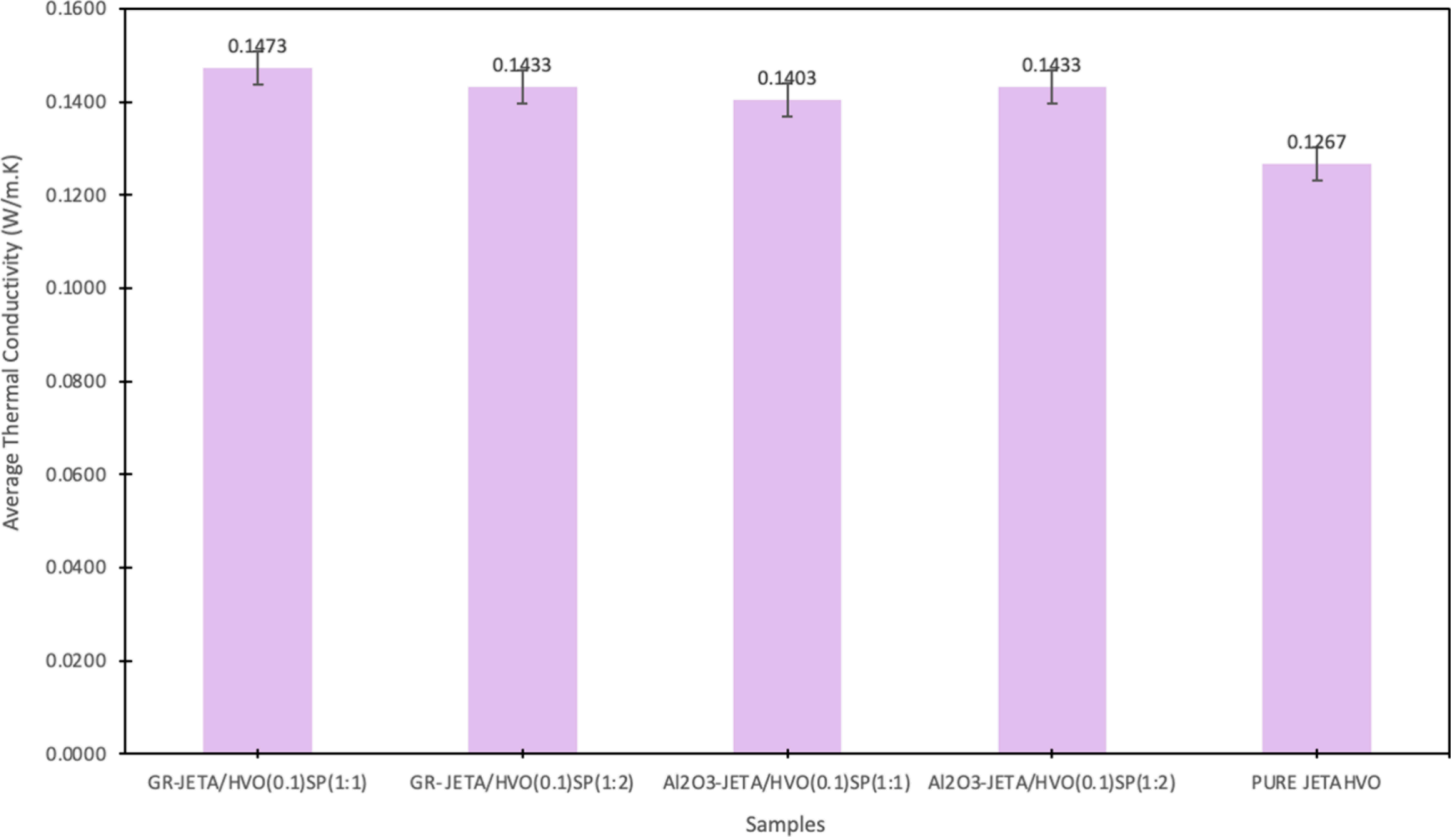
Graph of thermal conductivity of Gr and Al_2_O_3_ with Span 80 with 1:1 and 1:2 ratios and pure JetA-HVO.

As shown in Figure 7, the sample containing SPAN 80 and Gr at a 1:1 ratio exhibited a higher thermal conductivity than the sample with a 1:2 ratio, indicating that the optimal surfactant-to-nanoparticle proportion enhances thermal transport properties. This shows that the ratio of 1:1 of SPAN 80 with Gr is the most optimal in terms of thermal conductivity. This is because Gr is highly hydrophobic and has a large flat surface area. It interacts strongly with hydrophobic surfactants such as SPAN 80, allowing effective stabilization at a lower surfactant-to-nanoparticle ratio (1:1). Surfactant molecules can efficiently adsorb onto the graphene surface, providing steric stabilization and preventing aggregation. For the nonionic surfactant, increasing the surfactant concentration enhances the coverage of graphene surfaces, forming a steric barrier that prevents sheet agglomeration. Once the graphene surface is fully coated, further addition of surfactant produces negligible improvement in dispersion stability, as the sheets remain well-separated and uniformly suspended.^44^

In contrast, for Al_2_O_3_-based nanolubricants, the 1:2 ratio exhibited higher thermal conductivity than the 1:1 ratio, suggesting that increased surfactant concentration improves nanoparticle dispersion and enhances heat transfer performance. Similarly, Yang et al.^45^ investigated the optimum surfactant ratio for Al (alumina) nanoparticles with oleic acid in JP-10 fuel, a single-component advanced jet fuel, and reported comparable trends, with the 1:2 ratio providing the most effective dispersion. They concluded that the stability of nanofluids is directly related to the improvement in thermal conductivity; in other words, the observed increase in thermal conductivity was driven by improved surfactant behavior. Furthermore, Gr with surfactants was more thermally conductive than that of Al_2_O_3_. This improvement is due to the ability of carbon-based nanoparticles to enhance the thermal properties of nanolubricants.^46^ In combustion, effective heat transport leads to an even temperature distribution, thereby enhancing the fuel oxidation. Fuels or additives with higher thermal conductivity promote faster heat dissipation, allowing the flame to cool and burn more effectively.^47^ This enhancement allows the fuel droplets to heat up faster, which improves evaporation and atomization, which are two critical phases before combustion. The consequence is a shorter ignition delay and a greater combustion efficiency, which reduces pollutants such as CO and unburned hydrocarbons (UHCs).^48^

### 3.4 Zeta Potential

The ζ-potential test was used to determine the stability of colloidal dispersions. The ζ-potential refers to the electrostatic repulsion between particle surfaces and stationary fluid layers. Zeta potential values that are greater than +40 mV and less than −40 mV exhibit desirable stabilities.^36^

Figure 8 displays the average ζ-potential values of Al_2_O_3_ and Gr with SPAN 80 at 1:1 and 1:2 ratios to identify the optimal surfactant-to-nanoparticle ratio for stable nanofuels. On the basis of the average ζ-potential value, the results obtained for both the Gr and Al_2_O_3_ ratios align with the thermal conductivity findings, indicating that Al_2_O_3_ is more compatible at the 1:2 ratio, whereas the 1:1 ratio is optimal for Gr nanofuels. The average ζ-potential value for day 1 is higher compared to the ζ-potential value on day 3. The nanoparticles were anticipated to be uniformly dispersed within the base fluid immediately after preparation. These dispersion strategies inject energy into the system, facilitating the overcoming of van der Waals attractive forces that cause nanoparticles to aggregate.^49^ The average ζ-potential of all samples on day 3 was lower than that on day 1 because sedimentation occurred for all samples on day 3. According to the manual of Malvern Instruments,^50^ the amplitude of the zeta potential indicates the possible stability of the colloidal system. If all suspended particles possess a significantly negative or positive zeta potential, they will likely resist one another, resulting in no attraction or aggregation. However, if the particles exhibit low ζ-potential values, there will be insufficient force to inhibit their aggregation and/or flocculation. Furthermore, both ratios for the Gr nanofuels exhibited moderate stability compared with those of the Al_2_O_3_ nanofuels. The zeta potential value given for Al_2_O_3_ was lower than that of Gr. This finding is consistent with a study conducted by ref,^29^ which observed the zeta potential of Al_2_O_3_ in bio-oil with a concentration of 0.1 vol % and SDBS surfactant, yielding a zeta potential value of −2.76 mV. This study aims to examine the same trend as the current research, given the scarcity or near absence of investigations on Al_2_O_3_ in blended jet fuel with the specific surfactant, SPAN 80. The lower ζ-potential of Al_2_O_3_ is attributed to the metal oxide surfaces, which generally have a lower surface charge density and a narrower pH range in which they carry charge. Their ζ-potential can be closer to zero, especially near the isoelectric point, where the positive and negative charges balance each other out. In addition, a study^51^ reported the same result, with a slight decrease in the zeta potential as the graphene-to-surfactant ratio increased to 1:2.

**Figure 8:**
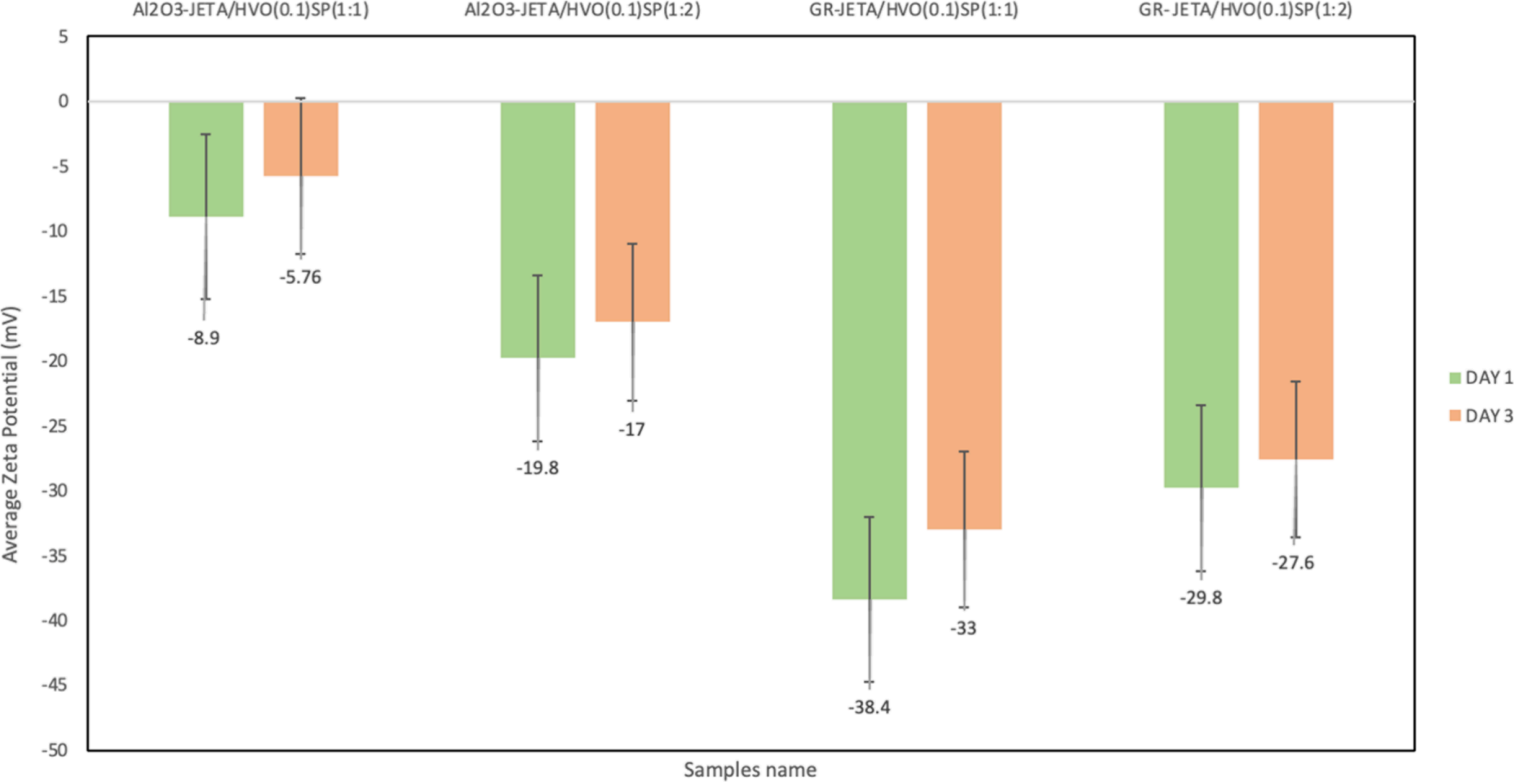
Average zeta potential of Al_2_O_3_ and Gr with Span 80 with a 1:1 and 1:2 ratio.

While this study used a 0.1 vol % nanoparticle concentration as a benchmark for aviation fuel additives, the concentration itself is critical to colloidal stability. In theory, a concentration of 0.1 vol % achieves a compromise between improving thermophysical characteristics and reducing particle collision frequencies. This balance is critical for sustaining the intended enhancements in performance while preserving the fuel stability and preventing engine blockage. Changing the nanoparticle concentration could have a major impact on the overall effectiveness of the additive in the aviation fuel. Higher concentrations, on the other hand, may cause increased viscosity and rapid sedimentation as the interparticle distance decreases. A greater reduction in concentration, on the other hand, may improve stability while reducing the returns on thermal conductivity improvements, which could lead to less efficient fuel performance under high-temperature conditions. The 0.1 vol % limit was chosen to preserve fuel atomization properties while investigating surfactant efficacy. Overall, the 0.1 vol % concentration provides a practical balance of stability and performance. It provides enhanced kinetic stability for up to 3 days, representing a significant improvement over the unsterilized blends while maintaining the necessary thermophysical qualities of the fuel. More research may be required to discover whether changes to the concentration could provide even larger advantages while maintaining the stability.

While the experimental results indicate that the optimal surfactant-to-nanoparticle ratios significantly reduce sedimentation compared with the base fuel, the observed stability period of approximately 3 days restricts long-term static storage in real aircraft applications. In this study, this duration is used as a comparative indicator of surfactant efficacy rather than a definitive answer for the long-term fuel shelf life. Fuel in actual aviation systems is subject to continual movement, vibration, and heat cycling, which may have an impact on precipitation kinetics. The 3 day window achieved here illustrates the difficulties of maintaining stable suspension in low-viscosity, nonpolar HVO/Jet-A blends, emphasizing the necessity for future research into high-energy sonication techniques or hybrid surfactant systems.

### 3.5 Study Limitations and Future Perspectives

Despite the significant discoveries on the interfacial effects of nonionic and anionic surfactants, this work acknowledges numerous limitations. The experiment was restricted to a constant nanoparticle concentration of 0.1 vol %. Subsequent studies should consider a broader concentration gradient, such as 0.01 to 0.5 vol %, to discover the ideal point at which stability and energy density are maximized. Furthermore, while stability was assessed at ambient temperatures, future research should look into the effects of high-pressure and high-temperature environments, simulating the conditions of an airplane fuel system. Investigating the synergistic effects of hybrid surfactants (nonionic and anionic combinations) remains a feasible strategy for achieving long-term suspension in complex SAF mixtures.

## 4 Conclusions

This study identifies the most suitable type and optimal ratio of surfactants for Gr and Al_2_O_3_ in blended Jet-A fuel with HVO to stabilize the nanofuels over a long period. The visual observation method was used for 7 days, showing that both Gr and Al_2_O_3_ were compatible with SPAN 80 at ratios of 1:1 and 1:2. Meanwhile, a 1:0.5 ratio of SPAN 80 was not optimal for either nanoparticle, as sedimentation was observed on day 1. Furthermore, CTAB was the least suitable surfactant for Gr and Al_2_O_3_, with nanofuels already sedimented on day 1 at all ratios. Thermal conductivity measurements revealed that Gr at a 1:1 ratio with SPAN 80 and Al_2_O_3_ at a 1:2 ratio exhibited the highest thermal conductivities. Gr is highly hydrophobic and has a large, flat surface area, which contributes to the low surfactant ratio needed to stabilize nanofuels. It strongly interacts with hydrophobic surfactants, such as SPAN 80, enabling effective stabilization at a lower surfactant-to-nanoparticle ratio (1:1). Additionally, the average ζ-potential for all nanofuels aligned with the thermal conductivity results, with Gr at a 1:1 ratio with SPAN 80 showing the highest ζ-potential at −38.4 mV, compared to −29.8 mV at a 1:2 ratio on day 1, indicating better stability at the higher surfactant ratio. Notably, elevating the surfactant concentration to a 1:2 ratio led to a reduction in the absolute ζ-potential to −29.8 mV. This indicates that at the 1:2 ratio, an excess of SPAN 80 molecules may have resulted in the formation of secondary micelles or a ‘depletion flocculation’ effect, wherein excess surfactant layer inhibits electrostatic repulsion and accelerates sedimentation. Meanwhile, the ζ-potential of Al_2_O_3_ at a 1:2 ratio with SPAN 80 was higher than that at a 1:1 ratio. Moreover, the higher ζ-potential value on day 1 compared to that on day 3 suggests that sedimentation on day 3 caused a decrease in the ζ-potential. The variation in nanoparticle types emphasizes the different properties of Gr and Al_2_O_3_, particularly regarding thermal conductivity and ζ-potential. Gr exhibits the highest values in both cases, owing to its carbon-based structure, which offers superior heat transfer capabilities compared to Al_2_O_3_. However, the current study shows that the identified surfactant with the specified ratio can only stabilize the nanofuel for 3 days. Further studies are needed to identify surfactants capable of stabilizing these nanoparticles in blended jet fuels for over a week or longer, ensuring their sustainable use in aeroengines that can be applied in real-life scenarios.

## Nomenclature

AlO: aluminum monoxide
Al_2_O_3_: aluminum oxide
CeO_2_: cerium oxide
CFA: carbon-rich fly ash
CNP: carbon nanoparticle
CTAB: Cetyltrimethylammonium bromide
CO: carbon monoxide
CO_2_: carbon dioxide
CuO: copper­(II) oxide
DLS: dynamic light scattering
DW: distilled water
EDL: electric double layer
EG: ethylene glycol
EU ETS: European Union Emissions Trading System
FAME: fatty acid methyl esters
FESEM: field emission scanning electron microscopy
Gr: graphene
HEFA: hydrotreated esters and fatty acids
HLB: hydrophobic–hydrophilic balance
HVO: hydrotreated vegetable oil
IEP: isoelectric point
KOH: potassium hydroxide
MWCNT: multiwalled carbon nanotubes
NOx: nitrogen oxide
NP: nanoparticle
PAG: polyalkylene glycol
PM: particulate matter
SAF: sustainable aviation fuel
SDBS: sodium dodecylbenzenesulfonate
SEM: scanning electron microscopy
Si: silicon
SiC: silicon carbide
Si_3_N_4_: silicon nitride
SiO_2_: silicon dioxide
SPAN 80: sorbitan monooleate
SWCNT: single-walled carbon nanotube
TiO_2_: titanium oxide
TEM: transmission electron microscopy
W/O: water-in-oil emulsion
ZnO: zinc oxide
ZP: zeta potential
